# Survey of Surgeon-reported Postoperative Protocols for Deep Inferior Epigastric Perforator Flap in Breast Reconstruction

**DOI:** 10.1097/GOX.0000000000005402

**Published:** 2023-11-15

**Authors:** Sthefano Araya, Madison Hackley, Grace M. Amadio, Mengying Deng, Civanni Moss, Eliann Reinhardt, Adam Walchak, Michael G. Tecce, Sameer A. Patel

**Affiliations:** From the * Division of Plastic and Reconstructive Surgery, Fox Chase Cancer Center, Philadelphia, Pa.; † Lewis Katz School of Medicine, Temple University, Philadelphia, Pa.; ‡ Biostatistics and Bioinformatics Facility, Fox Chase Cancer Center, Philadelphia, Pa.; § Albany Medical College, Albany, N.Y.

## Abstract

**Background:**

The use of deep inferior epigastric perforator (DIEP) flaps is a well-established breast reconstruction technique.

**Methods:**

A 29-question survey was e-mailed to 3186 active American Society of Plastic Surgeons members, aiming to describe postoperative monitoring practice patterns among surgeons performing DIEP flaps.

**Results:**

From 255 responses (8%), 79% performing DIEP surgery were analyzed. Among them, 34.8% practiced for more than 20 years, 34.3% for 10–20 years, and 30.9% for less than 10 years. Initial 24-hour post-DIEP monitoring: intensive care unit (39%) and floor (36%). Flap monitoring: external Doppler (71%), tissue oximetry (41%), and implantable Doppler (32%). Postoperative analgesia: acetaminophen (74%), non-steroidal anti-inflammatory drugs (69%), neuromodulators (52%), and opioids (4.4%) were administered on a scheduled basis. On postoperative day 1, 61% halt intravenous fluids, 67% allow ambulation, 70% remove Foley catheter, and 71% start diet. Most surgeons discharged patients from the hospital on postoperative day 3+. Regardless of experience, patients were commonly discharged on day 3. Half of the surgeons are in academic/nonacademic settings and discharge on/after day 3.

**Conclusions:**

This study reveals significant heterogeneity among the practice patterns of DIEP surgeons. In light of these findings, it is recommended that a task force be convened to establish standardized monitoring protocols for DIEP flaps. Such protocols have the potential to reduce both the length of hospital stays and overall care costs all while ensuring optimal pain management and vigilant flap monitoring.

**Takeaways**

**Question:** What are the postoperative protocol practices of surgeons performing deep inferior epigastric perforator (DIEP) flaps?

**Findings:** A survey of active American Society of Plastic Surgeons members revealed heterogeneity in the practice patterns of DIEP surgeons. Variability was observed in postoperative care settings, monitoring techniques, timing of monitoring, analgesia, postoperative care protocols, and discharge dates.

**Meaning:** These findings highlight the need for improvement and standardization to enhance patient outcomes and optimize the cost–benefit relationship.

## INTRODUCTION

For over 25 years, deep inferior epigastric perforator (DIEP) flap postoperative protocols for breast reconstruction have displayed considerable variation among surgeons in monitoring, discharge, analgesia, and techniques.^1^ Multiple flap monitoring techniques have been described in the literature, including tissue oximetry’s high sensitivity for pedicle-related issues.^2^ However, the literature has supported that clinical monitoring remains the superior monitoring technique for flaps utilized in breast reconstruction.^3^ There is no statistically significant difference in false-negative rates or flap salvage rates between clinician monitoring and the use of an implantable Doppler.^3^ Although Enhanced Recovery After Surgery (ERAS) advises that frequent postoperative monitoring with clinical evaluation is often sufficient, implantable Doppler devices are recommended for buried flaps.^4^ Pain management is pivotal, and combined analgesia proves effective and reduces opioid consumption after breast reconstruction surgery.^5,6^ ERAS also advocates multimodal opioid-sparing regimens.^4^

This study provides an objective characterization of current national trends and the persistent heterogeneity among active plastic surgeons in DIEP flap protocols, despite the presence of ERAS guidelines, while also highlighting the need for standardization of ERAS protocols.

## METHODS

A 29-question cross-sectional, anonymous survey on DIEP flap monitoring protocols was e-mailed to a random cohort of 3186 active American Society of Plastic Surgeons (ASPS) surgeon members. Institutional review board approval for this survey study was obtained under institutional review board number 21-8014. The survey was first deployed via e-mail on April 19, 2022, and was deployed for a second time on June 22, 2022. The survey officially closed on July 7, 2022. The survey was accessible through e-mail from April through July 2022. The survey included questions on surgeons’ practice patterns for various aspects of flap management, postoperative flap monitoring setting, length of stay, postoperative analgesic use, and monitoring techniques. Demographic information related to the respondents’ length of practice and the type of practice setting was also collected. (**See survey, Supplemental Digital Content 1**, which displays the final survey that was used, http://links.lww.com/PRSGO/C857). All the responses collected from the survey were analyzed using Microsoft Excel.

## RESULTS

### Surgeon Characteristics

Among respondents, 34.8% practiced for over 20 years; 34.3%, between 10 and 20 years; and 30.9%, under 10 years. Practice settings were academic (26.5%), solo (26.5%), group (32.5%), and employed (14.5%). Of the surgeons, 63% performed one to five procedures per month; 22%, six to 10 per month; and 14%, 10 to 20 per month. Only 24% devoted 100% to reconstructive surgery; 36% allocated 75% to reconstruction. Others balanced cosmetic and reconstructive cases in varied proportions.

### Monitoring Techniques

**Fig. 1.**
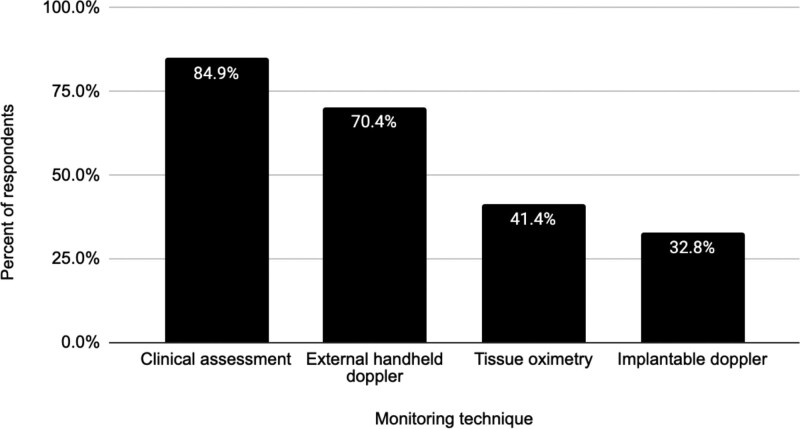
Percentage of postoperative monitoring techniques used by respondents.

**Fig. 2.**
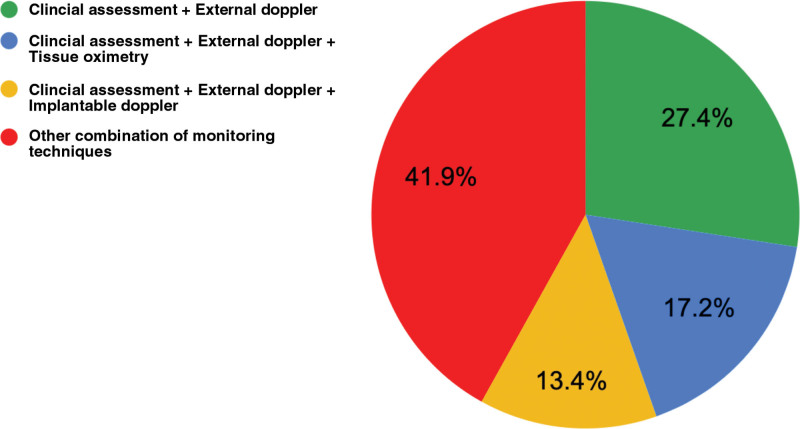
Percentage of common monitoring technique combinations by respondents.

Active ASPS members primarily use clinical assessment (84.9%) and external handheld Doppler (70.4%) for DIEP flap monitoring. Tissue oximetry and implantable Doppler are employed by 41.4% and 32.8%, respectively (Fig. 1). The most prevalent combination is clinical assessment + external handheld Doppler (27.4%). The next common method involves clinical assessment, external handheld Doppler, and tissue oximetry (17.2%). A combination of clinical assessment, external handheld Doppler, and implantable Doppler is employed by 13.4% (Fig. 2).

### Postoperative Setting

**Fig. 3.**
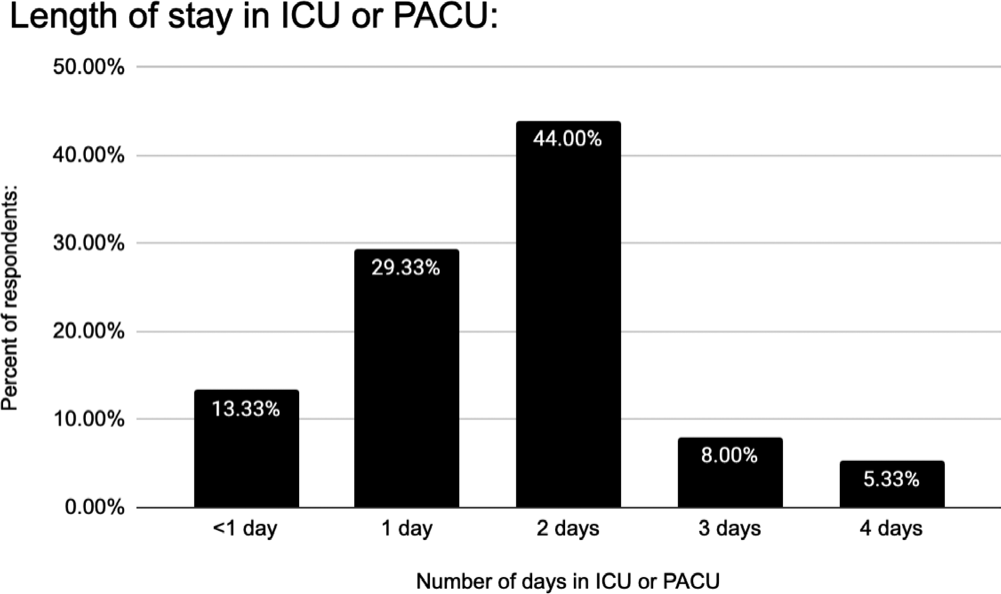
Length of time before patients monitored in the PACU or ICU are transferred to the regular floor for monitoring.

**Fig. 4.**
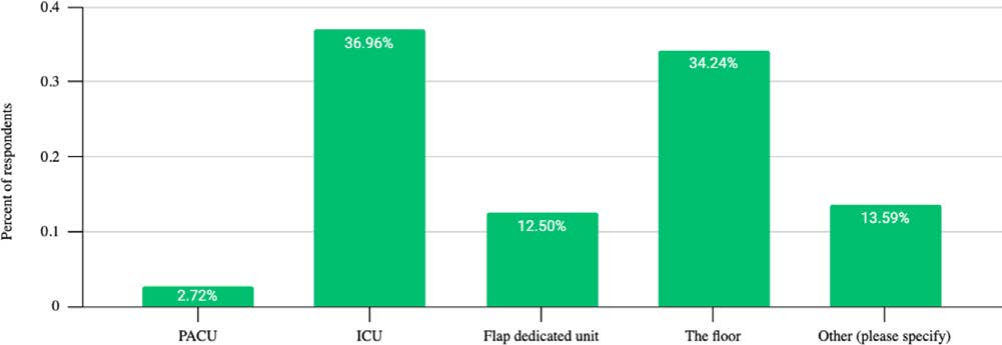
Postoperative DIEP flap monitoring setting first 24 hours.

Intensive care unit (ICU) (39%) primarily monitors DIEP flap cases within 24 hours. For stay duration, 44% of respondents reported a 2-day ICU stay before the floor (Fig. 3). The floor (36%) directly receives patients postsurgery (Fig. 4).

**Fig. 5.**
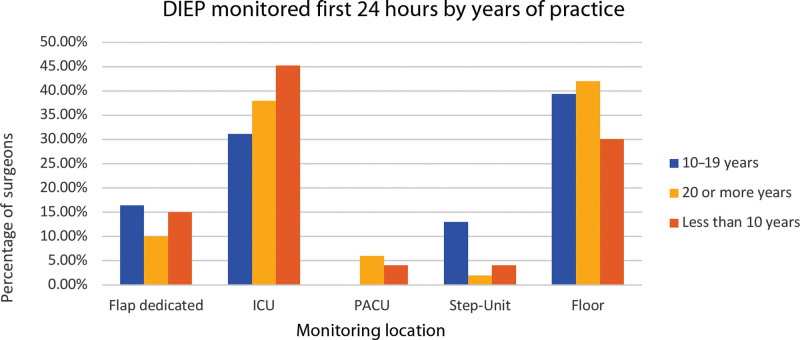
DIEP flap monitoring first 24 hours by years of practice.

**Fig. 6.**
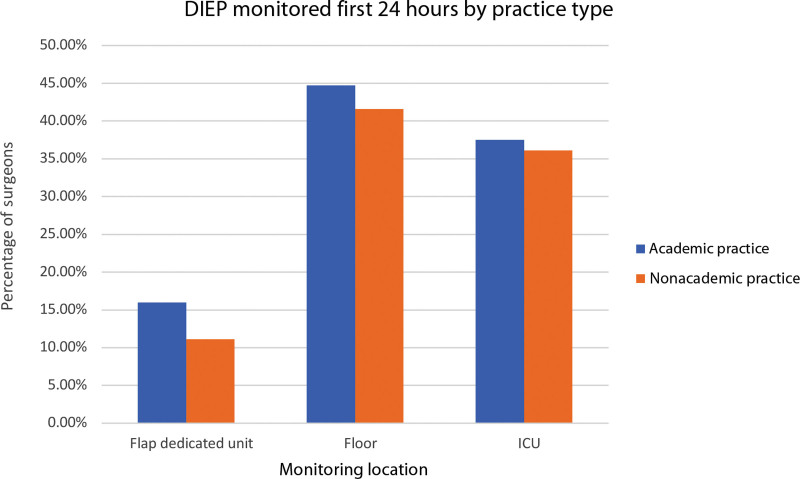
Principal DIEP Flap monitoring first 24 hours by practice setting.

Experience-wise, flap monitoring was done as follows: over 20 years—floor (42%), ICU (38%), dedicated flap floor (10%), and post-anesthesia care unit (PACU)/step-down (10%); under 10 years—ICU (45%), floor (30%), dedicated flap floor (12%), and PACU/step-down (10%). from 11 to 19 years—floor (39%), ICU (31%), dedicated flap floor (16%), and PACU/step-down (14%) (Fig. 5). Regarding practice settings, flap monitoring was done as follows: academic floor (45%) and ICU (38%), versus nonacademic floor (42%), and ICU (36%) (Fig. 6).

### Flap Check Pattern

Of respondents, 42.7% checked flaps hourly on postoperative day (POD) 1, 17.2% on POD 2, and only 2.1% on POD 3. On POD 2, 27.9% checked every 2 hours. Up to POD 3, 31.2% checked every 4 hours, and 9.4% checked every 4 hours up to POD 4.

### Opioid Usage

**Fig. 7.**
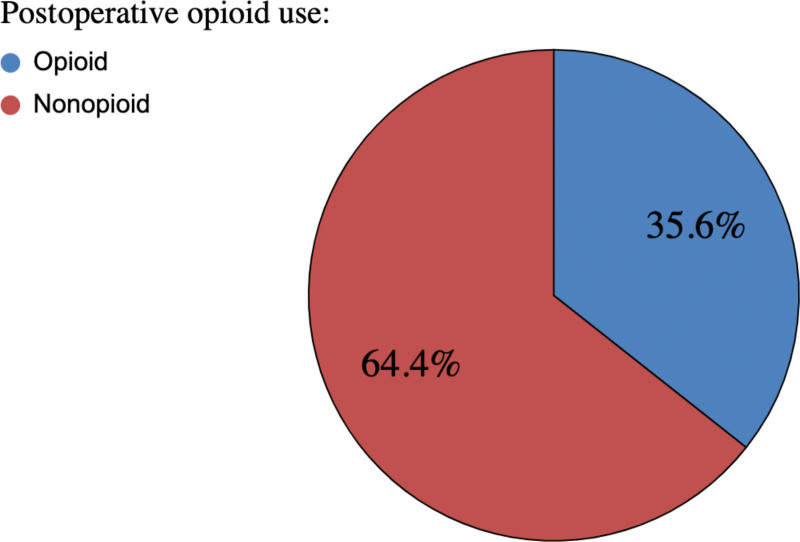
Postoperative analgesic use: opioid vs nonopioid pain control.

**Fig. 8.**
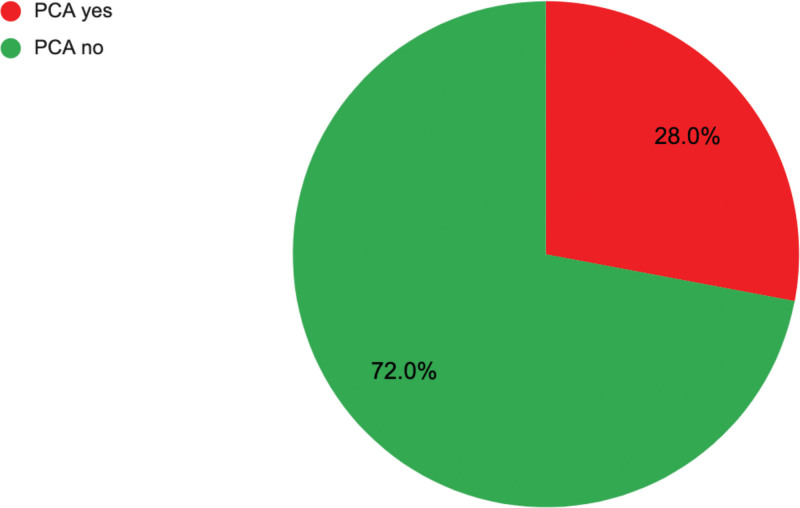
Postoperative PCA use.

**Fig. 9.**
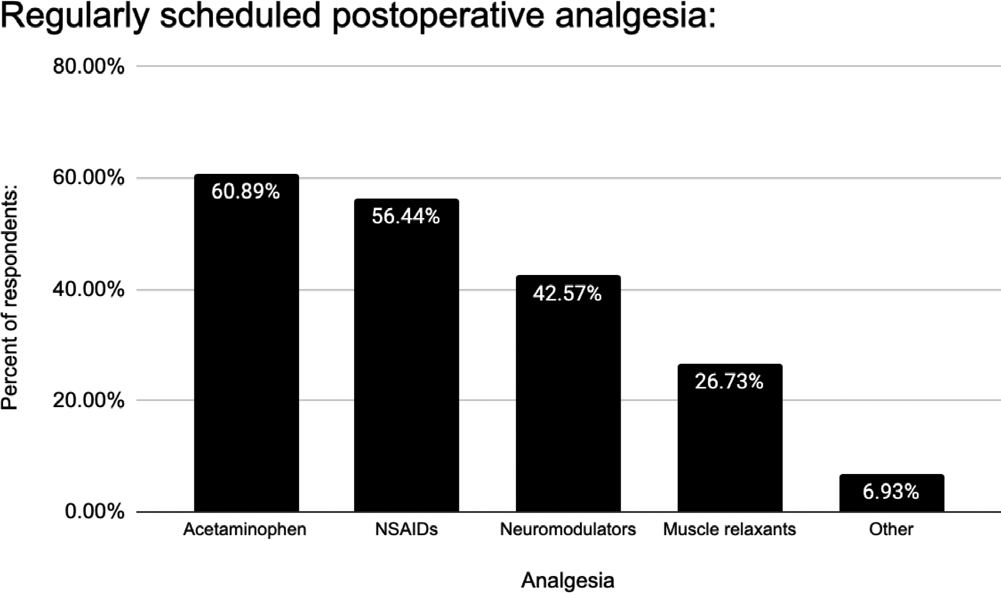
Postoperative analgesic use: general categories.

Fifty-eight respondents (31.5%) used opioids for pro re nata (PRN) postoperative pain control (Fig. 7). Twenty-eight percent used patient-controlled analgesia (PCA) pumps for postoperative narcotics (Fig. 8). Notably, only 74% employed acetaminophen (Tylenol), 56% used non-steroidal anti-inflammatory drugs (NSAIDs), 42% used neuromodulators, and 26% used muscle relaxants for standing postoperative analgesia (Fig. 9). Only 49% of respondents employed a combination of acetaminophen and NSAIDs (15%), or this combination with another pain control method (34%). Acetaminophen and NSAIDs alone were used for PRN postoperative analgesia 4% and 5% of the time, respectively. Just 10% used both acetaminophen and NSAIDs PRN postoperatively.

### Discharge Protocols

**Fig. 10.**
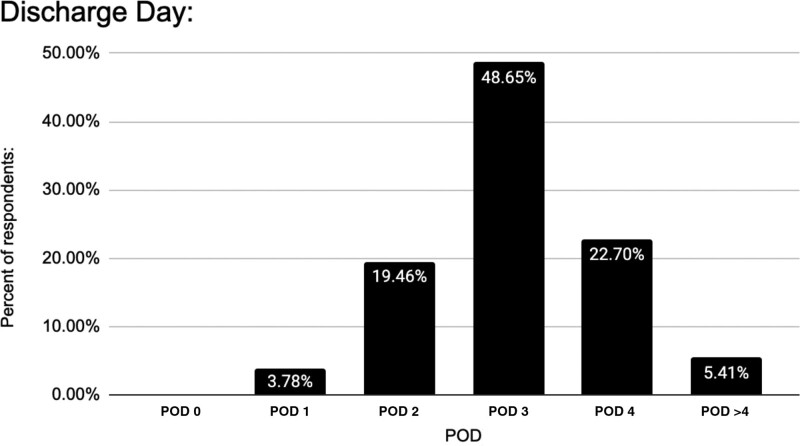
Percentage of patients discharged on PODs 0–4+.

Describing discharge planning, 62% of respondents cease postoperative chemoprophylaxis before hospital discharge. On POD 1, 61% halt intravenous (IV) fluids, 67% initiate patient ambulation, 70% remove Foley catheters, and 71% commence regular diets. On POD 2, 27% halt IV fluids, 21% encourage patient ambulation, 20% remove Foley catheters, and 19% start regular diets. Yet, only 19.5% usually discharge on POD 2. Common discharge dates are POD 3 (49%) and POD 4 (23%) (Fig. 10).

**Fig. 11.**
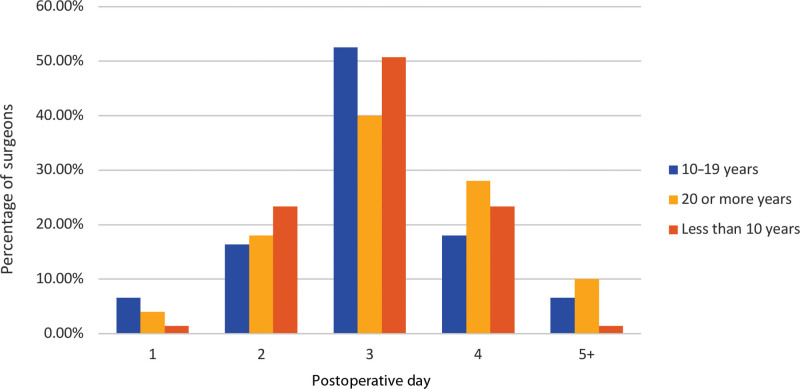
Typical postoperative discharge day by years of practice.

Across experience groups, day 3 discharge is common (40%, 53%, and 51% for 20+, 11–19, and ≤10 years’ experience, respectively). Surgeons with 10 years of experience or less discharged earlier (23% on day 2 and 1.4% on day 1). Experienced surgeons (20+ and 11–19 years) have lower day 2 rates (18% and 16%, respectively). For 20+ years, 28% discharge on day 4, and 10% beyond; 11–19 years, 18% on day 4, and 6.6% beyond; 10 years or less, 23% on day 4, and 1% beyond (Fig. 11). Overall, day 3 discharge is common. Younger surgeons discharge earlier; experienced lean to day 4+.

**Fig. 12.**
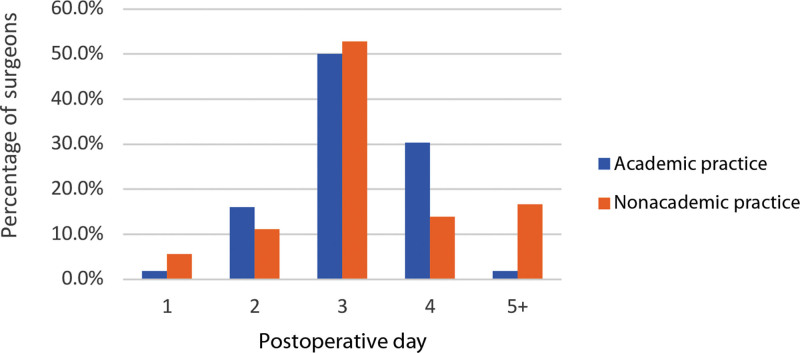
Typical postoperative discharge day by practice type.

Practice setting influences were as follows: academic—50% day 3, 30% day 4, and 17% day 2; nonacademic—53% on day 3, 14% on day 4, and 11% on day 2 (Fig. 12). Both favor day 3 to 4, and academic slightly prefers day 4.

## DISCUSSION

### Postoperative Setting

Varied postoperative settings for DIEP flap breast reconstruction emerged, primarily favoring the ICU or floor. Our survey reveals that 37% of surgeons use ICU monitoring for the first 24 hours and 43% continue until POD 2 (Fig. 4). The survey’s postoperative monitoring setting diversity underscores the lack of a unified recommendation. The ICU represents a costly flap monitoring setting for a generally noncritical, stable patient. Vemula et al found no significant difference in outcomes between ICU-monitored tertiary care facilities and non-ICU specialty surgery hospitals. Flap success rates (98.22% and 98.81%) also showed no significant difference.^7^

With 15 years of experience, our institution efficiently monitors patients on the floor via trained nurses and an ERAS protocol. Our publication recommends floor flap monitoring, depending on the practice setting. Our study showcases reduced stays and unchanged complications/flap failures because of our ERAS protocol implementation on the floor.^8^

Surgeons should optimize cost-effective and adequate monitoring while balancing available resources. Ochoa et al, through an ERAS pathway, achieved this in their private practice, reducing the length of stay and ICU use.^9^

Our survey shows that 26.5% of respondents are in academic practice, whereas the rest are in private settings (Fig. 6). Concerns about applying this trend to private practice have previously been described. Establishing a dedicated flap floor for monitoring in private practice could be challenging due to differing financial and labor resources compared with academic centers, posing standardization hurdles.^9^ Adopting ERAS and standard monitoring for both settings is vital. Yet, assessing their unique needs/resources is also vital for success.

In our high-volume academic center, our nursing staff receives continuous exposure and education, ensuring that the majority of nurses are well trained and confident in monitoring flaps. Considering our institution’s data and prior studies, this survey suggests a shift from ICU monitoring to a regular floor. A regular floor with experienced nursing offers monitoring without excessive costs or interventions, preserving ICU access for critical cases. ASPS task force–developed ERAS protocols could address this, potentially reducing stays, saving costs, and standardizing care for patients undergoing DIEP flap reconstruction.

### Flap Monitoring Techniques

Flap monitoring techniques for DIEP flap breast reconstruction patients varied broadly among ASPS respondents. A combination of clinical assessment and external handheld Doppler was the most common technique (25%) (Fig. 1). ERAS protocol considers Doppler an extension of clinical assessment due to being “noninvasive, inexpensive, and widely used.”^4^ A combination of clinical assessment, external handheld Doppler, and tissue oximetry was the second most common (15.8%) (Fig. 2). Additionally, 12.4% of respondents used a varied combination including clinical assessment, external handheld Doppler, and an implantable Doppler. The ERAS protocol consensus for microvascular breast reconstruction states that clinical assessment (temperature, color, capillary refill, and external handheld Doppler) is sufficient on its own in those cases where there is an external skin paddle.^4^ There is no statistically significant difference in false-negative rates or flap salvage rates between clinician monitoring and the use of implantable Doppler.^3^

Only 25.2% of respondents employ ERAS-recommended monitoring techniques. External handheld Doppler and clinical assessment remain reliable with trained healthcare personnel. Technologies such as tissue oximetry and implantable Doppler benefit less experienced teams, aiding early flap compromise detection, especially in buried flaps. Although tissue oximetry identifies adverse deoxygenation events with high sensitivity, the challenge lies in weighing early detection against frequent false alarms.^2^ Balancing costs against salvage benefits, particularly in low-flap-failure institutions, is essential.^10^ Although no guideline is suggested, tissue oximetry is noninvasive and cost-effective when used selectively, despite potential false positives. Estimated potential savings are up to $1667 per DIEP flap procedure.^11^ However, technology adoption depends on various practice setting variables, including baseline take-back rates, resident and fellow monitoring support, and nursing comfort and competence in flap monitoring, among others.

### Flap Check Timing

Postoperative monitoring timing for DIEP flaps varies among ASPS respondents. Although the ERAS protocol recommends hourly checks in the first 24 hours, every 2 hours for the next 24 hours, and every 3–4 hours thereafter, survey results reveal deviations.^12^ Despite this guideline, the survey results show that 19.3% of respondents were still conducting hourly flap checks on both POD 2 (17.2%) and POD 3 (2.1%). In total, 27.9% of respondents adhered to the ERAS protocol by conducting checks every 2 hours on POD 2.^4^ Moreover, 40.6% conducted 4-hourly checks on POD 3 (31.2%) and POD 4 (9.4%), aligning with ERAS recommendations.^4^

This timing variability, despite consensus recommendations, leads to unnecessary monitoring at times, increasing costs without added benefit. Baltodano et al’s analysis of national data highlighted reduced reoperation rates comparing POD 2 and POD 1.^13^ Their data supports the majority of takebacks and close monitoring every hour (Q1), and every two hours (Q2) would be most beneficial in the first 2 PODs, respectively. For most cases, focusing on these initial 2 days could improve the cost-effectiveness relationship, with closer monitoring reserved for higher-risk patients.

### Opioid Analgesia

Opioid-limited management minimizes postoperative complications such as nausea, vomiting, and constipation, promoting early mobilization.^4^ Adhering to the ERAS protocol’s opioid-limited approach enhances mobility, shortens hospital stays and does not increase complications.^12^ However, 31.5% of ASPS surgeon respondents use opioids for as-needed postoperative analgesia (Fig. 7), with 28% administering postoperative IV PCA narcotics (Fig. 8), indicating ongoing reliance on opioids for postoperative pain relief. Acetaminophen and nonsteroidal anti-inflammatory agents are utilized as PRN postoperative analgesia 51.6% and 47.3% of the time, respectively (Fig. 9). Notably, only 9.4% use both despite evidence showing that their combined effectiveness surpasses that of acetaminophen alone.^4^

The most prevalent preoperative and postoperative analgesic combinations are NSAIDs and neuromodulators (16.8% and 20.8%, respectively). Moreover, 24.5% use neuromodulators (such as gabapentin and pregabalin) alone, whereas 52.5% rely solely on muscle relaxants (cyclobenzaprine, diazepam, or methocarbamol). These findings underscore the need for improved evidence-based postoperative analgesia practice standardization.

In line with the ERAS Society Consensus, combining acetaminophen and NSAIDs alongside other modalities is recommended for optimal postoperative pain control. Achieving opioid-sparing analgesia aligns with ERAS goals of reducing postoperative nausea, vomiting, and constipation, facilitating early patient mobilization.^4^ Although many respondents use acetaminophen and NSAIDs PRN, considering their synergistic benefits, we suggest incorporating them as standing postoperative analgesic orders.^4^

Prior studies like those of Sindali et al and Rendon et al observed substantial opioid reduction with ERAS protocols utilizing nonopioid analgesics after DIEP flap procedures.^14,15^ At our institution, implementing an ERAS protocol incorporating preoperative and postoperative nonopioid analgesia led to PCA pump elimination, further supporting effective opioid reduction.^8^

Strong evidence underlining opioid-limited management’s potential to curtail complications emphasizes the significance of pain management protocols prioritizing reduced opioid use. Moreover, limited narcotics not only correlate with fewer complications but also result in shorter hospital stays, indirectly driving cost savings.

The findings of Ochoa et al in a private practice setting mirrored this trend. Their ERAS protocol implementation yielded decreased narcotic use between pre-ERAS and post-ERAS groups.^9^ Strong evidence underlining opioid-limited management’s potential to curtail complications emphasizes the significance of pain management protocols prioritizing reduced opioid use. Moreover, limited narcotics not only correlate with fewer complications but also result in shorter hospital stays, indirectly driving cost savings.

Survey responses and previous studies together should urge surgeons to enhance their protocols, focusing on decreasing opioid usage by prioritizing non-narcotic pain management as the foundation for analgesia. This approach can lead to shorter hospital stays and cost reduction. The adoption of standardized standing orders for multimodal, nonopioid postoperative pain control should become a widespread practice among surgeons.

### Discharge Protocols/ERAS

Clear protocols for determining patient stability for discharge according to ERAS are lacking. The current protocol discusses thromboembolism prophylaxis for “higher” risk patients, as defined by the Caprini risk assessment model, until the patient is ambulatory or discharged.^4,16^ Regarding venous thromboembolism (VTE) prophylaxis, 35.3% of ASPS survey respondents endorsed using the Caprini risk method to determine anticoagulation necessity, but 62% discontinued chemoprophylaxis upon patient discharge. Valuable literature offers diverse views on deep vein thrombosis prophylaxis. ASPS recommends VTE prophylaxis for Caprini scores above 3, with prolonged prophylaxis (4 weeks) for scores exceeding 7.^17^ Given this recommendation, it would be expected that most, if not all, ASPS surgeons would use the Caprini score to determine VTE prophylaxis for their patients. However, surprising findings indicate a majority of surgeons discontinue prophylaxis upon the patient’s discharge from the hospital.

This finding aligns with Huang et al, who reviewed 249 patient charts. The cohort’s average Caprini score was 6.0, with 72.7% between 3 and 6, and 26.5% at 7 or higher. Interestingly, only one patient (0.4%) with a score of 7, receiving in-hospital prophylaxis, developed deep vein thrombosis; no pulmonary embolism cases occurred. Notably, VTE rates did not significantly differ between guideline-consistent and nonconsistent chemoprophylaxis.^17^

Conversely, Modarressi et al found in a similar study that over 90 PODs, four patients (2.1%) had pulmonary embolism, and two patients (1%) developed deep venous thrombosis, yielding an overall VTE incidence of 3.1%. Interestingly, most patients (92.2%) had high-risk Caprini scores (>5), and all VTE cases were within this group. Besides the Caprini score, no specific risk factor was identified, emphasizing the score’s value.^18^

Only 35% of surgeons use this tool, whereas 62% discharge patients without VTE prophylaxis. Bridging this gap and promoting broader Caprini score adoption could enhance patient outcomes and decrease VTE incidence in high-risk cases.

Most patients are discharged on POD 3 (49%) or POD 4 (22%) (Fig. 10). This trend is consistent across experience groups (20+, 11–19, and <10 years) with day 3 being the primary choice, followed by day 4 at 28%, 18%, and 23%, respectively (Fig. 11). However, younger surgeons tend to discharge earlier, whereas more experienced ones lean toward day 4 or later. By practice setting, POD 3 is preferred (Fig. 12). Notably, the majority discontinue IV fluids, enable ambulation, remove the Foley catheter, and initiate a regular diet on POD 1. Postdischarge supportive services were not analyzed in this survey.^7^

The collected data indicate that patients undergoing DIEP flap surgery often meet discharge benchmarks (Foley removal, normal diet, IV discontinuation, ambulation) a day or two before actual discharge, implying safe earlier discharge if clinically clear and indicated. Our institution, after implementing the DIEP ERAS protocol, has achieved an average length of stay of 2.91 days.^8^ Supported by referenced literature, previous studies support aiming for earlier discharge without compromising outcomes or increasing complications.^8,9,13,19–25^

### Cost

Prior research highlights a significant cost reduction with just a 1-day discharge date reduction.^22–25^ Jablonka et al found that surpassing the $100,000/quality-adjusted life year threshold for society’s willingness to pay, the cost-utility of inpatient flap monitoring beyond POD 2 is evident. For instance, saving a failing flap on POD 3 costs $2,154,751 for 907 flaps while monitoring 121 flaps on POD 0–1 costs only $287,610.^22^ This underscores the highest flap failure risk within the initial two PODs, diminishing cost-effectiveness beyond this window.^13,22^

Understanding the current practice patterns for DIEP flap monitoring is crucial for optimizing healthcare costs and hospital resources. Recent research comparing ERAS protocol-based DIEP flap surgery to nonprotocol surgery demonstrated lower patient costs ($25,915 versus $30,709) and enhanced hospital profitability potential with ERAS implementation.^26^

O’Neill et al corroborated the cost benefits of shortened hospital stays, achieving a decrease from 4.7 to 3.6 days (*P* = 0.001) with ERAS protocol adoption. This change led to a 28% cost reduction for unilateral cases and 24% for bilateral cases—equivalent to around CAD $881 (US $665) and CAD $838 (US $632) per day of postoperative care, respectively.^24^ Similarly, Kaoutzanis et al accomplished a 1.7-day reduction in length of stay through ERAS implementation, translating to a $4400 per-patient saving and estimated overall savings of $279,258, including increased contribution margins of $189,342.^25^ Both studies achieved cost savings while maintaining similar outcomes and complication rates.

Nonetheless, our survey unveiled that 28.81% of respondents discharge DIEP flap patients on POD 4 or later, which contrasts with recent safe early discharge recommendations. Enhancing ERAS and discharge protocols could enhance cost-effectiveness without compromising patient care, ensuring suitable patients receive safe and earlier discharges.

### Limitations

Although a higher response rate would have been preferable, the attained 8% response rate provides valuable insights from a subset of DIEP flap-performing plastic surgeons. However, we recognize that this lower response rate could introduce response bias and impact the findings’ applicability to the entire ASPS surgeon population. We emphasize the significance of aiming for improved response rates in upcoming survey-based research to bolster the results’ reliability and broader relevance.

## CONCLUSIONS: A CALL TO ACTION

Results highlight significant DIEP flap monitoring heterogeneity, with diverse care settings, techniques, timing, analgesia, protocols, and discharge dates. This variation suggests room for standardization and enhancement.

Considering widespread ERAS protocol support, including in DIEP flap surgery, a workgroup should establish a unified ERAS protocol. Central to intervention effectiveness is the concept of healthcare value, where optimizing outcomes against costs plays a pivotal role. The literature robustly advocates for standardized ERAS DIEP protocols, aligning with healthcare’s pursuit of greater value through enhanced results and reduced expenses.

## DISCLOSURES

The authors have no financial interest to declare in relation to the content of this article. This study was funded by NIH/NCI grant P30CA006927 (Fox Chase Cancer Center Support Grant). A Smiles Factory grant funded the survey deployment.

## Supplementary Material


