# Supplementary material for: Survey of Surgeon-reported Postoperative Protocols for Deep Inferior Epigastric Perforator Flap in Breast Reconstruction
Source: Plast Reconstr Surg Glob Open. 2023 Nov 15;11(11):e5402. doi: 10.1097/GOX.0000000000005402 (PMC10653572; doi:10.1097/GOX.0000000000005402)
Supplement: Supplementary file 1 [file gox-11-e5402-s001.pdf]

DIEP Flap Monitoring Protocols Survey

*** 1. Do you perform DIEP breast reconstruction surgery?**

- ◯ Yes
- ◯ No

AMERICAN SOCIETY OF PLASTIC SURGEONS®

DIEP Flap Monitoring Protocols Survey

Your completion of the survey or questionnaire will serve as your consent to be in this research study.

Please read all questions and choose the answer that best reflects the policies and procedures of your institution.

2. For how many postoperative days do you typically perform Q1 hour flap checks?

- Postoperative day 0
- Postoperative day 1
- Postoperative day 2
- Postoperative day 3
- Postoperative day 4
- Postoperative day >4
- N/A

3. For how many postoperative days do you typically perform Q2 hour flap checks?

- Postoperative day 0
- Postoperative day 1
- Postoperative day 2
- Postoperative day 3
- Postoperative day 4
- Postoperative day >4
- N/A

4. For how many postoperative days do you typically perform Q4 hour flap checks?

- Postoperative day 0
- Postoperative day 1
- Postoperative day 2
- Postoperative day 3
- Postoperative day 4
- Postoperative day >4
- N/A

*** 5. At your institution, where are DIEP flaps monitored for the first 24 hours?**

- PACU
- ICU
- Flap dedicated unit
- The floor
- Other (please specify)

6. If your patients spend part of their recovery after surgery at PACU or ICU, how many days (counting the first 24 hours) do your patients stay there before being transferred to the regular floor?

- <1 day
- 1 day
- 2 days
- 3 days
- 4 days
- 5 days
- > 5 days
- N/A - Patient goes to the floor immediately after surgery.

7. What does monitoring of the flap consist of? (Select all that apply)

- [ ] No monitoring
- [ ] Clinical assessment (temperature, color, capillary refill)
- [ ] External handheld Doppler
- [ ] Other (please specify)
- [ ] ViOptix (tissue oximetry)
- [ ] Implantable Doppler

8. How do you use antibiotics as part of your DIEP postoperative Protocol?

- ( ) Preoperatively
- ( ) Perioperatively for 23 hours or less
- ( ) Throughout hospital admission
- ( ) Throughout hospital admission and PO after discharge
- ( ) Other (please specify)

9. Do your patients typically receive any of the following blocks? (Select all that apply)

- [ ] TAP block
- [ ] Thoracic paravertebral
- [ ] Epidural
- [ ] Other (please specify)
- [ ] PECS1
- [ ] PECS2

10. Which of these medications do you regularly use as PRN preoperative analgesia? (Select all that apply)

- [ ] Acetaminophen
- [ ] NSAIDs
- [ ] Neuromodulators (gabapentin, pregabalin)
- [ ] Muscle relaxants (i.e. cyclobenzaprine, diazepam)
- [ ] Other (please specify)

11. Which of these medications do you regularly use as PRN postoperative analgesia? (Select all that apply)

- [ ] Acetaminophen
- [ ] NSAIDs
- [ ] Neuromodulators (gabapentin, pregabalin)
- [ ] Muscle relaxants (i.e. cyclobenzaprine, diazepam)
- [ ] Other (please specify)

12. Which of these medications do you regularly use as scheduled preoperative analgesia? (Select all that apply)

- [ ] Acetaminophen
- [ ] NSAIDs
- [ ] Neuromodulators (gabapentin, pregabalin)
- [ ] Muscle relaxants (i.e. cyclobenzaprine, diazepam)
- [ ] Other (please specify)

13. Which of these medications do you regularly use as scheduled postoperative analgesia? (Select all that apply)

- [ ] Acetaminophen
- [ ] NSAIDs
- [ ] Neuromodulators (gabapentin, pregabalin)
- [ ] Muscle relaxants (i.e. cyclobenzaprine, diazepam)
- [ ] Other (please specify)

14. Do your patients get a postoperative narcotic IV PCA?

- Yes
- No

15. Do you routinely give your patients preoperative chemoprophylaxis (i.e. unfractionated Heparin or low molecular weight heparin) as part of your DIEP preoperative Protocol?

- Yes
- No

16. Do you give your patients postoperative chemoprophylaxis (i.e. unfractionated Heparin or low molecular weight Heparin) as part of your DIEP postoperative Protocol?

- Yes
- No

17. What is your metric for discontinuation of postoperative chemoprophylaxis?

- Patient is ambulating
- Patient is discharged from the hospital
- First 2 weeks post-discharge
- Longer than 2 weeks post-discharge (please specify)

18. Do you use therapeutic systemic anticoagulation postoperatively as part of your DIEP Protocol?

- Yes
- No

19. When do you usually discontinue IV fluids?

- Postoperative day 0
- Postoperative day 1
- Postoperative day 2
- Postoperative day 3
- Postoperative day 4
- Postoperative day >4

20. When are your patients allowed to ambulate?

- Postoperative day 0
- Postoperative day 1
- Postoperative day 2
- Postoperative day 3
- Postoperative day 4
- Postoperative day >4

21. When is the Foley catheter typically removed?

- Postoperative day 0
- Postoperative day 1
- Postoperative day 2
- Postoperative day 3
- Postoperative day 4
- Postoperative day >4

22. When do you start your patients on a clear liquid diet?

- Postoperative day 0
- Postoperative day 1
- Postoperative day 2
- Postoperative day 3
- Postoperative day 4
- Postoperative day >4
- N/A - Patients start with regular diet

23. When do you start your patients on a regular diet (solid food by mouth)

- Postoperative day 0
- Postoperative day 1
- Postoperative day 2
- Postoperative day 3
- Postoperative day 4
- Postoperative day >4

24. When are your patients usually discharged from the hospital?

- Postoperative day 0
- Postoperative day 1
- Postoperative day 2
- Postoperative day 3
- Postoperative day 4
- Postoperative day >4

25. On average, how many DIEP flap surgeries do you perform per month?

- ◯ 1-5
- ◯ 6-10
- ◯ 11-15
- ◯ 16-20
- ◯ >20

26. On average, how many DIEP flap surgeries does your institution perform per month?

- ◯ 1-5
- ◯ 6-10
- ◯ 11-15
- ◯ 16-20
- ◯ >20

27. How many years have you been in practice?

- Less than 5
- 5 – 9
- 10 – 14
- 15 – 19
- 20 – 24
- 25 years or more

28. What best describes your practice type?

- Solo practice
- Solo practice-shared facility
- Small plastic surgery group practice (2-5 plastic surgeons)
- Employed Physician
- Large plastic surgery group practice (6 or more plastic surgeons)
- Medium multi-specialty group practice (6-20 physicians)
- Large multi-specialty group practice (more than 20 physicians)
- Academic practice
- Academic practice (salaried with private practice)
- Employed Physician
- Military
- Retired

29. Please indicate which of the following best describes how you practice in terms of TIME spent:

- 100% Reconstructive
- Approximately 25% Cosmetic and 75% Reconstructive
- Approximately 50% Cosmetic and 50% Reconstructive
- Approximately 75% Cosmetic and 25% Reconstructive
- Approximately 100% Cosmetic

Finished

Thank you for taking time to complete this survey. Your input is extremely valuable to us.

If you have any questions or comments, please contact us at research@plasticsurgery.org
